# Solvent Fractionation Improves the Functional Properties of Sheep Rump Fat: Effects of Different Lipid Fractions on Lipid Metabolism and Gut Health in Mice

**DOI:** 10.3390/foods14213641

**Published:** 2025-10-24

**Authors:** Xin Ma, Junfei Yu, Zequan Xu, Jian Wei, Lingyan Wu, Hongjiao Han, Jianzhong Zhou, Zirong Wang

**Affiliations:** 1 College of Food Science and Pharmacy, Xinjiang Agricultural University, Urumqi 830052, China; maxin913@xjau.edu.cn (X.M.); yu13837808512@163.com (J.Y.); x624173842@xjau.edu.cn (Z.X.); w0l9y19@126.com (L.W.); 13998311554@163.com (H.H.); 2 School of Food Science and Technology, Shihezi University, Shihezi 832003, China; weijian@shzu.edu.cn

**Keywords:** Altay sheep rump fat, solvent fractionation, fatty acid composition, blood lipid metabolism, gut microbiota, short-chain fatty acids

## Abstract

To enhance the nutritional value of sheep fat, high-melting-point solid fat (HSO) and low-melting-point liquid oil (LSO) were prepared from Altay sheep rump fat via solvent fractionation. The effects of HSO and LSO on lipid metabolism and intestinal health were evaluated in a mouse model. Results showed that HSO, rich in saturated fatty acids (SFA), induced obesity, dyslipidemia, and colonic inflammation in mice. These adverse effects were associated with the upregulation of hepatic lipid synthesis genes such as Sterol regulatory element-binding protein 1c (*SREBP-1c*) and Fatty acid synthase (*FAS*), as well as increased expression of pro-inflammatory cytokines including Tumor necrosis factor-alpha (TNF-α) and Interleukin-6 (IL-6) in the colon. In contrast, LSO, which was predominantly composed of unsaturated fatty acids (UFA), did not cause significant metabolic disorders. Instead, it promoted the upregulation of fatty acid oxidation-related genes such as Peroxisome proliferator-activated receptor alpha (*PPARα*) and Acyl-CoA oxidase 1 (*Acox1*), helped maintain intestinal microbial balance, and enhanced the production of beneficial short-chain fatty acids (SCFAs), particularly butyrate and propionate. In conclusion, solvent fractionation effectively modulates the fatty acid composition of sheep fat, thereby influencing lipid metabolism and inflammatory responses through the regulation of key gene expression and modulation of the gut microenvironment.

## 1. Introduction

In physiological processes, animal fats play a dual role: they serve not only as essential sources of energy and regulators of physiological functions [1], but their composition and molecular structure also profoundly influence human health [2]. Animal fats such as cattle fat and sheep fat occupy a prominent position in the food industry due to their distinctive flavors and favorable processing properties. However, these fats are rich in saturated fatty acids and cholesterol; long-term consumption may increase the risk of hyperlipidemia, hypertension, and other metabolic disorders, thereby posing a serious threat to human health [3]. Oil fractionation technology has emerged as a key strategy for improving the quality of animal fats by exploiting differences in triglyceride melting points. Through controlled cooling and crystallization, this process enables physical separation into solid and liquid phases [4], yielding products with tailored melting behaviors and enhanced stability [5]. Solvent fractionation—achieved by mixing oils with organic solvents such as acetone, followed by temperature-controlled crystallization—allows for the isolation of solid fats and liquid oils with distinct thermal properties. Salas et al. [6] successfully produced high-stearic sunflower butter using this method as a cocoa butter substitute, while Kapseu et al. [7] confirmed the efficacy of acetone in facilitating efficient oil fractionation. However, existing research has predominantly focused on the fractionation of vegetable oils and milk fats, with insufficient attention paid to the fractionation of sheep fat—a species-specific lipid with a unique fatty acid profile. Moreover, these studies have largely been confined to process optimization and basic physicochemical characterization, failing to elucidate the intrinsic relationship between fractionated components and host metabolism. This gap represents a critical and unexplored area in the field.

Previous studies have demonstrated that lipid fractions with different melting points can modulate obesity development through mechanisms involving digestion, absorption, and lipid metabolism regulation [8]. High-melting-point fats may reduce energy availability due to lower digestibility, whereas low-melting-point oils exert beneficial effects by modulating lipoprotein metabolism and mitigating inflammatory responses [9]. Although these findings have established a preliminary link between fat melting point and health effects, the conclusions are primarily derived from studies on fractionated products of palm oil or milk fat. Sheep fat exhibits a distinct specificity in its fatty acid composition, which suggests that the metabolic pathways and health impacts of its fractionated components may differ markedly. Currently, there is a lack of systematic investigation addressing this critical distinction. Moreover, obesity development is closely linked to dietary fat intake and gut microbiota composition. High-fat diets can disrupt microbial homeostasis, altering the Firmicutes/Bacteroidetes ratio and promoting increased energy harvest and adiposity [10]. Therefore, the current research gap specifically lies in the unclear mechanism of how fractionated sheep fat components systemically influence host health via the “blood lipid metabolism–gut microbiota” axis. Elucidating this mechanism constitutes the core scientific question addressed by the present study.

This study focuses on the rump fat of Altay sheep, a native breed in Xinjiang, China, known for its high nutritional value. Altay sheep rump fat constitutes approximately 17% of carcass weight at slaughter [11], representing an abundant fat source. Our prior work revealed significant differences in fatty acid profiles among fractionated components of sheep rump fat [12], and preliminary animal trials indicated that these fractions enhance hepatic antioxidant enzyme activity and reduce oxidative stress—findings that challenge the conventional notion that animal fat intake invariably leads to lipid dysregulation [13]. These preliminary findings suggest that fractionated sheep fat components may play a distinct role in regulating host health. Based on this, the present study aims to systematically compare and analyze the differential effects of Altay sheep rump fat and its fractionated components on host lipid metabolism and gut microbiota structure through well-controlled animal experiments. By doing so, it seeks to elucidate the underlying mechanism by which fractionated sheep fat influences host health via the “blood lipid metabolism–gut microbiota” axis. This work will lay a groundwork for future studies aimed at further understanding the relationship between animal fat and host health.

## 2. Materials and Methods

### 2.1. Materials

Sheep rump fat (from 15-month-old male Altay sheep, commercially slaughtered in Xinjiang) and cattle fat (from 5-year-old male Xinjiang brown cattle, commercially slaughtered) were purchased from Hualing Market, Urumqi, Xinjiang, China. Rapeseed oil (Fortune brand) was purchased from Youhao Supermarket, Urumqi, Xinjiang, China.

Preparation of crude sheep rump fat: An appropriate amount of sheep rump fat was heated in a water bath at 100 °C for 2 h until completely melted, with continuous stirring during the process. The melted fat was then cooled to 60 °C, filtered through double-layer gauze to obtain crude sheep rump fat, and stored at −25 °C for later use.

Preparation of fractionated products: Based on the optimized solvent fractionation process for sheep rump fat established in our previous study [14], a measured amount of crude sheep rump fat was placed in an SSW-600-2S electric constant-temperature water bath (Boxun, Shanghai, China) and heated to 60 °C until complete melting was achieved. Acetone was then added at a 1:2 (*v*/*v*) ratio relative to the fat, and the mixture was thoroughly homogenized by shaking. The solution was subsequently transferred to a SPX-150 biochemical incubator (Jiangsu Jinyi Instrument Technology Co., Ltd., Changzhou, China) and cooled at a controlled rate of 6 °C/h to a final temperature of 8 °C. After 16 h of crystallization, the mixture was centrifuged at 4000 r/min for 15 min using a 4-20R refrigerated centrifuge (Heng-n, Changsha, China) to separate the high-melting-point solid fat (HSO) from the low-melting-point liquid oil (LSO). The resulting fractions were then transferred to a DZF vacuum drying oven (Hengnuo Lixing Technology Co., Ltd., Beijing, China) and dried under vacuum conditions (70 °C, 0.08 MPa) for 4 h to completely remove residual acetone. Following GB 5009.262-2016, the acetone residue was determined and found to be below the limit of quantification in both fractionated products, indicating effective removal of the solvent.

### 2.2. Physicochemical Indicator Analysis

The melting point was determined according to GB/T 24892-2010 [15] “Determination of Melting Point (Sliding Point) of Animal and Vegetable Fats and Oils in Open Capillary Tubes.” The iodine value was measured following GB/T 5532-2022 [16] “Determination of Iodine Value of Animal and Vegetable Oils.” Malondialdehyde (MDA) content was quantified using an ELISA kit based on the method described by Liu J et al. [17]. Peroxide value was determined according to GB 5009.227-2023 [18], acid value according to GB 5009.229-2016 [19], and color parameters (L*, a*, b*) were measured using an ADCI-60-C automatic colorimeter following the method described by Liu et al. [20].

Methylation was performed according to the procedure of Rebechi [21] with minor modifications. Briefly, 0.1 g of oil was accurately weighed into a test tube, followed by the addition of 5 mL of *n*-hexane. The mixture was vortexed for 5 min to ensure complete dissolution. Subsequently, 2 mL of 0.5 mol/L potassium hydroxide-methanol solution was added, thoroughly mixed, and incubated in a 60 °C water bath for 30 min to complete the transesterification reaction. After standing at room temperature for 1 h, the mixture separated into two phases, and the upper organic layer was filtered through a 0.22 μm organic membrane to remove particulate impurities. Fatty acid composition was analyzed following the method of Henmi [22] using a 7890B gas chromatograph (Agilent, Palo Alto, CA, USA) equipped with a fused-silica capillary column (ANPEL Cd-2560, Shanghai, China; 100 m × 0.25 mm i.d., 0.25 µm film thickness). Nitrogen was used as the carrier gas. The injection port temperature was set at 240 °C with a split ratio of 100:1, and the injection volume was 1 μL. The oven temperature program began at 140 °C for 5 min, increased at 4 °C/min to 240 °C, and held for 30 min. The detector (Flame ionization detector, FID) was maintained at 250 °C, with hydrogen flow at 30 mL/min, air at 300 mL/min, and nitrogen make-up gas at 30 mL/min. Fatty acids were identified by comparing retention times with a standard mixture of 37 fatty acid methyl esters (Source Leaf Biotechnology Co., Ltd., Shanghai, China), and their relative abundances were quantified using the area normalization method. Each sample was analyzed in triplicate, and the mean value was reported as the final result. We performed independent preparation and triplicate measurements (*n* = 3) for each oil sample. All values are reported as mean ± standard deviation.

### 2.3. Experimental Animals and Diet

A total of 60 male C57BL/6 mice weighing 20 ± 2 g were provided by the Animal Experiment Center of Xinjiang Medical University. The experimental protocol was approved by the Animal Welfare Ethics Committee of Xinjiang Agricultural University (Approval No. 2024031). Mice were housed under controlled conditions: temperature around 24 °C, 12 h light/dark cycle, and relative humidity of 50–60%. After 7 days of acclimation, the mice were randomly assigned to six groups (*n* = 10 per group): blank control (CK, gavaged with equivalent physiological saline), rapeseed oil (RO), cattle fat (CA), crude sheep rump fat (SO), high-melting-point solid fat (HSO), and low-melting-point liquid oil (LSO). The gavage experiment lasted for 40 days.

All mice had free access to a standard chow diet (composition shown in Table 1), with the gavaged oils provided as an additional fat source not included in the basal diet fat content. The gavage dose was determined based on the average daily edible oil intake of Chinese residents (43.2 g/person/day) [23]. Using a standard human body weight of 60 kg and a human-to-mouse dose conversion factor of 0.91, the calculated gavage dose for mice was 0.13 mg/20 g body weight. The CK, RO, and LSO groups received oils in liquid form at room temperature, while CA, SO, and HSO were melted in a 45 °C water bath and cooled to near body temperature before gavage. Body weight was measured every 5 days to adjust the gavage volume accordingly. After 40 days, following a 12 h fast (with free access to water), the mice were weighed, euthanized, and samples were collected for subsequent analysis.

### 2.4. Sample Collection

Serum sample collection: Blood samples were collected via orbital sinus puncture and transferred into centrifuge tubes. The samples were centrifuged at 4 °C at 4000 rpm for 8 min, and the resulting supernatant was collected as serum. Serum samples were stored at −80 °C in a DW-86L630 vertical ultra-low temperature freezer (Qingdao Haier Group Co., Ltd., Qingdao, China) for subsequent analysis. Liver tissue collection: Immediately after euthanasia, liver tissues were excised on a sterile operating table and rinsed with normal saline for subsequent analysis. Colon tissue collection: Following euthanasia, colon and cecal contents were collected on a sterile operating table. The contents were rapidly frozen in liquid nitrogen for subsequent analysis of gut microbiota and SCFAs. The colon tissue was rinsed with normal saline and fixed in 5% formalin solution for 24 h before histological examination.

### 2.5. Determination of Growth Indicators

Body weight and body length measurement: Body weight and body length were measured every three days before gavage, and the initial and final body weights and body lengths of mice in each group were recorded.

Body mass index (BMI) measurement: The BMI of mice was calculated using the following formula:

BMI (g/cm^2^) = body weight (g)/length^2^ (cm^2^) (1)

Lee index (Lee’s) measurement: The Lee index of mice was calculated using the following formula:

Lee’s = cube root of body weight (g)/length (cm)(2)

Organ coefficient determination: Immediately after euthanasia, the liver, spleen, and kidneys were rapidly excised. Surface blood was carefully blotted with filter paper, and the weight of each organ was measured individually. The organ coefficient was then calculated using the following formula.

Organ coefficient (%) = [organ weight (g)/body weight (g)] × 100(3)

### 2.6. Determination of Blood Lipids and Histopathological Examination of Colon Tissues

Serum samples were processed according to the protocols of the Nanjing Jiancheng Bioengineering Institute assay kits, and the levels of total cholesterol (TC), triglycerides (TG), low-density lipoprotein cholesterol (LDL-C), and high-density lipoprotein cholesterol (HDL-C) in mice were measured using an Xmark microplate reader (Bio-Rad, Hercules, CA, USA). The atherogenic index (AI) was calculated using the following formula:

AI = (TC − HDL-C)/HDL-C(4)

Histopathological Observation: Colon tissues were fixed in 5% neutral formaldehyde solution for 24 h, followed by gradient dehydration, wax immersion embedding, block trimming, sectioning, dewaxing, rehydration, staining, dehydration, clearing, and mounting. Prepared tissue sections were observed and photographed under a CX21 ordinary optical microscope (Olympus, Tokyo, Japan), an IX51 inverted white light/fluorescence photography microscope (Olympus, Tokyo, Japan), and a CX31 upright white light photography microscope (Olympus, Tokyo, Japan). The histological scoring criteria for colonic injury were established according to the method described by Majumder et al. [24], as detailed in Supplementary Table S1.

### 2.7. Real-Time Quantitative Polymerase Chain Reaction (qPCR)

Frozen liver tissue (0.1 g) was homogenized in 1 mL of TRIzol reagent using grinding beads. After centrifugation, the supernatant was transferred to an RNase-free tube, mixed with 200 μL of chloroform, and centrifuged. The aqueous phase was collected, combined with an equal volume of isopropanol, and incubated for 10 min to precipitate RNA. The pellet was washed with a DEPC–ethanol (1:3) solution and finally dissolved in DEPC-treated water. RNA was reverse-transcribed into Complementary deoxyribonucleic acid (cDNA) using the RR047A kit (TaKaRa, Bio Inc., Dalian, China) according to the manufacturer’s instructions. Quantitative PCR was performed using SYBR Green Realtime PCR Premix under the following conditions: 95 °C for 30 s, followed by 40 cycles of 95 °C for 5 s and 60 °C for 30 s. The relative mRNA expression levels in mouse liver were calculated by the 2^−∆∆Ct^ method. Glyceraldehyde-3-phosphate dehydrogenase (GAPDH) and Beta-actin (β-actin) were used as reference genes for inflammatory factor-related genes and lipid metabolism-related genes, respectively. Primer sequences were designed based on nucleotide sequences obtained from the NCBI database, using Primer 6 and Oligo 7 software in accordance with standard primer design principles. All primers were synthesized by Sangon Biotech (Shanghai, China) Co., Ltd., and are listed in Supplementary Table S2.

### 2.8. Gut Microbial Analysis

#### 2.8.1. PCR Amplification and 16S rDNA Sequencing

Primers were designed based on conserved regions of microbial sequences, with sample-specific Barcode sequences added. The variable regions of rRNA genes were amplified by PCR using an ABI GeneAmp^®^ 9700 PCR amplifier (Beijing Cybergene Technology Co., Ltd., Beijing, China). Primers 341F (CCTAYGGGRBGCASCAG) and 806R (GGACTACNNGGGTATCTAAT) were selected, with the reaction program as follows: initial denaturation at 98 °C for 1 min, followed by 30 cycles of 98 °C (10 s), 50 °C (30 s), and 72 °C (30 s), ending with a 5 min extension at 72 °C. PCR products were purified using magnetic beads, mixed in equal amounts according to their concentrations, and the target bands were detected and recovered after thorough mixing. Library preparation was completed through steps including end repair, A-tailing, sequencing adapter ligation, and purification. The constructed library was quantified using Qubit and qPCR, and paired-end sequencing was performed on an Illumina Miseq high-throughput sequencer (Illumina, San Diego, CA, USA) based on the Illumina sequencing platform.

#### 2.8.2. Biological Analysis

Raw data were filtered to remove low-quality reads and chimeric sequences, yielding effective sequences for operational taxonomic unit (OTU) clustering. Using QIIME2 (Version 2024.10) software (Northern Arizona University, Flagstaff, AZ, USA), sequences were denoised and taxonomically annotated against the Silva database (Max Planck Institute, Berlin, Germany). Finally, data from all samples were normalized for subsequent analysis.

### 2.9. Extraction and Analysis of SCFAs

The extraction of SCFAs was performed following the method of Mahdi et al. [25]. with appropriate modifications. Briefly, the sample was accurately weighed and placed into a 2 mL centrifuge tube. Subsequently, 1 mL of ultrapure water was added, and the mixture was vortexed for 10 s. Stainless steel grinding beads were then added, and the sample was processed in a JXFSTPRP-24 cryogenic grinding instrument (Shanghai Jingxin Technology Co., Ltd., Shanghai, China) at a frequency of 40 Hz for 4 min. The sample was then placed in a PS-60AL ultrasonic instrument (Maikeyi (Beijing) Technology Co., Ltd., Beijing, China) immersed in an ice water bath and ultrasonicated for 5 min. This grinding-ultrasonication cycle was repeated three times. Following centrifugation at 4 °C and 5000 rpm for 20 min using a Fresco17 centrifuge (Thermo Fisher Scientific, Waltham, MA, USA), 0.8 mL of the supernatant was transferred to a new centrifuge tube. Next, 0.1 mL of 50% sulfuric acid (*v*/*v*) (Sinopharm Group, Beijing, China) and 0.8 mL of methyl tert-butyl ether extraction solution containing 25 mg/L 2-methylvaleric acid as an internal standard (Shanghai Anpu Experimental Technology Co., Ltd., Shanghai, China) were added sequentially. The mixture was vortexed for 10 s, followed by horizontal shaking for 10 min. The sample was then ultrasonicated in an ice water bath for 10 min. After centrifugation at 4 °C and 10,000 rpm for 15 min, the supernatant was stored at −20 °C for 30 min prior to instrumental analysis. The quantification of SCFAs was conducted according to the method of Han et al. [26], using a GC-2030 gas chromatograph (Agilent, Palo Alto, CA, USA) equipped with an HP-FFAP column (30.00 m × 250.00 μm × 0.25 μm) and a QP2020 NX mass spectrometer (Shimadzu Corporation, Tokyo, Japan). The carrier gas was high-purity helium at a constant flow rate of 1.2 mL/min, with an injection volume of 1 μL and a split ratio of 5:1. The septum purge flow rate was set to 3 mL/min. The temperature program was as follows: initial temperature of 50 °C for 1 min, ramped to 150 °C at 50 °C/min and held for 1 min, then to 170 °C at 10 °C/min with no hold time, followed by an increase to 240 °C at 25 °C/min and held for 1 min, and finally to 40 °C/min with a 1 min hold. The injection port temperature was maintained at 220 °C, the ion source (electron ionization mode) at 240 °C, the ionization energy was 70 eV, and the quadrupole temperature was set to 150 °C. Cecal content samples were collected from five randomly selected mice per group as biological replicates (*n* = 5). For each biological replicate, three technical replicates were analyzed, and the results are expressed as mean ± standard deviation.

### 2.10. Data Processing

All experimental data are expressed as mean ± standard deviation. Statistical analysis was performed using one-way analysis of variance (ANOVA) in SPSS 27.0 (Stanford University, Stanford, CA, USA). Effect sizes for ANOVA were reported as η^2^ and interpreted as follows: η^2^ < 0.01, trivial; 0.01 ≤ η^2^ < 0.06, small; 0.06 ≤ η^2^ < 0.14, moderate; and η^2^ ≥ 0.14, large. The significance level was set at α = 0.05. For all multiple hypothesis testing, the Benjamini–Hochberg method was applied to control the false discovery rate (FDR), with an FDR-corrected *p* < 0.05 considered statistically significant. Differences in microbial community structure based on Bray–Curtis distances were assessed using Adonis (PERMANOVA) with *p* < 0.05 indicating significance. All figures were generated using Origin 2024 (OriginLab Corp., Northampton, MA, USA).

## 3. Results

### 3.1. Physicochemical Property Analysis

**Figure 1:**
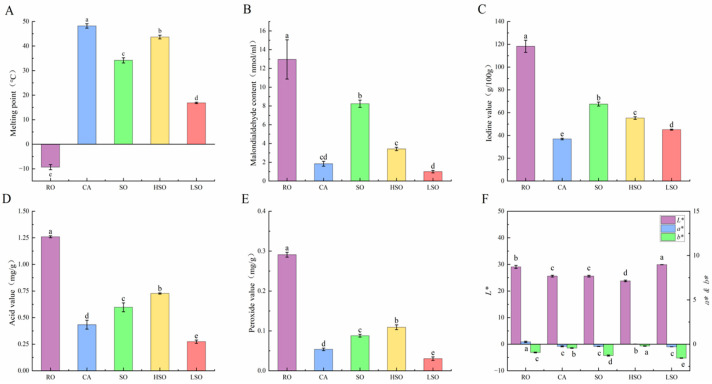
Physicochemical indices of different oils and fats. (**A**) Melting points, (**B**) MDA, (**C**) Iodine value, (**D**) Acid value, (**E**) Peroxide values, and (**F**) Color parameter analysis, The L*, a*, and b* values represent lightness, red-green, and blue-yellow, respectively. Results are presented as mean ± standard deviation (*n* = 5). Different lowercase letters above the bars indicate significant differences between groups (*p* < 0.05).

To investigate the differences in physicochemical properties among various oils, we determined the melting point, malondialdehyde (MDA) content, iodine value, acid value, peroxide value, and color parameters. As shown in Figure 1A, significant differences (*p* < 0.05) were observed in the melting points of the oils, ranked in descending order as CA > HSO > SO > LSO > RO. CA exhibited the highest melting point (48.20 °C), while RO showed the lowest (−9.33 °C). MDA, a product of lipid oxidation, reflects the degree of oil oxidation. RO contained the highest MDA level (12.96 nmol/mL), whereas LSO had the lowest (1.00 nmol/mL) (Figure 1B). Compared with SO, both HSO and LSO obtained by fractionation showed significantly reduced MDA contents (*p* < 0.05). Iodine values also differed significantly among the oils (*p* < 0.05, Figure 1C), with the order RO > SO > HSO > LSO > CA. The highest iodine value was observed in RO (118.22 g/100 g), and the lowest in CA (36.94 g/100 g). Acid values (Figure 1D) were highest in RO (1.26 mg/g) and lowest in LSO (0.27 mg/g), showing significant differences among groups (*p* < 0.05). Similarly, peroxide values (Figure 1E) were highest in RO (0.29 mg/g) and lowest in LSO (0.03 mg/g), with significant inter-group variation (*p* < 0.05). Color parameter analysis (Figure 1F) revealed that LSO had the highest L* value, RO showed the highest a* value, and HSO exhibited the highest b* value.

### 3.2. Fatty Acid Analysis

The major fatty acid composition of the different oils is presented in Table 2. CA exhibited the highest SFA content (65.54%), which was significantly greater than that of all other groups (*p* < 0.05). Conversely, RO showed the lowest SFA (6.01%) and the highest UFA content (88.41%), which was predominantly composed of monounsaturated fatty acids (MUFA, 59.64%) and polyunsaturated fatty acids (PUFA, 28.77%), mainly oleic acid (C18:1n-9c) and linoleic acid (C18:2n-6c).

Among sheep rump fat and its fractions, the SFA content in LSO (36.05%) was significantly lower than that in SO (43.91%), while its UFA content (56.32%) was significantly higher than that in both HSO (32.27%) and SO (48.67%), with oleic acid being the predominant MUFA. Furthermore, the PUFA content in LSO (11.04%) was also significantly higher than that in HSO (7.63%) and SO (9.97%). Solvent fractionation effectively modified the fatty acid profile of sheep rump fat, resulting in significant SFA enrichment in HSO and a notable increase in UFA in LSO.

### 3.3. Growth and Organ Coefficient Indicators

**Figure 2:**
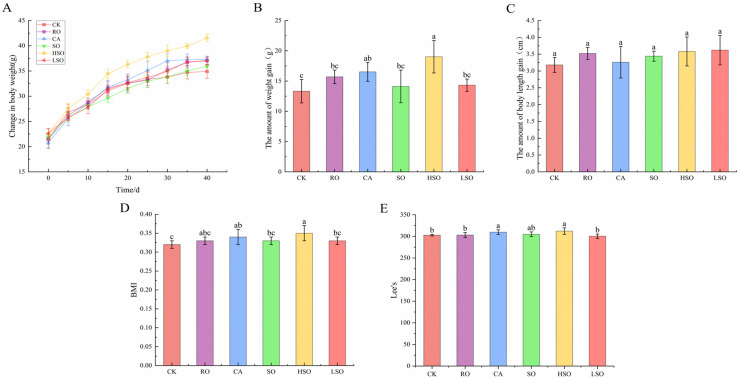
Growth indicators in mice: (**A**) changes in body weight, (**B**) increase in body weight, (**C**) increase in body length, (**D**) BMI, and (**E**) Lee’s index. Results are presented as mean ± standard deviation (*n* = 5). Different lowercase letters above the bars indicate significant differences between groups (*p* < 0.05).

Changes in body weight, body length, BMI, and Lee’s index of mice are shown in Figure 2. After 40 days of gavage, there was a highly significant difference in weight gain among the groups (ANOVA: F = 5.643, *p* = 0.001), with an η^2^ value of 0.540, indicating a large effect size. The CA and HSO groups showed the greatest increases in body weight. The weight gain in the CK group was 13.32 g, and the HSO group had a significantly higher weight gain than the CK group (*p* < 0.05), while the RO, SO, and LSO groups showed no significant difference compared to CK (*p* > 0.05). No significant difference was observed in body length gain among the groups (ANOVA: F = 1.375, *p* = 0.275, η^2^ = 0.220). Although the η^2^ value suggested a large effect size, the limited sample size (*n* = 30) may have resulted in insufficient statistical power to reliably confirm this effect. Significant differences were found in both BMI (ANOVA: F = 3.218, *p* = 0.023, η^2^ = 0.401) and Lee’s index (ANOVA: F = 3.594, *p* = 0.014, η^2^ = 0.480), with both effect sizes being large. Compared to the CK group (BMI: 0.32, Lee’s index: 302.82), the HSO group exhibited significantly higher values (BMI: 0.35, Lee’s index: 312.27; *p* < 0.05). The CA group also had a significantly higher BMI than the CK group (*p* < 0.05), while no significant differences were observed in BMI or Lee’s index between the other groups and CK (*p* > 0.05). Among all groups, the LSO group showed the lowest BMI (0.33) and Lee’s index (300.46). Detailed ANOVA results are provided in Supplementary Table S3.

**Figure 3:**
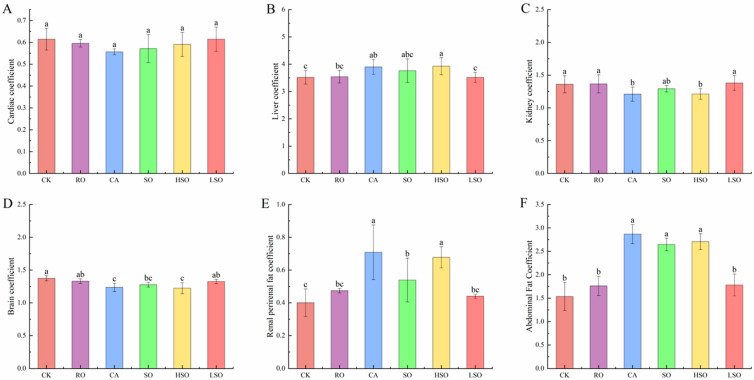
Organ indices in mice: (**A**) heart coefficient, (**B**) liver coefficient, (**C**) kidney coefficient, (**D**) brain coefficient, (**E**) perirenal fat coefficient, and (**F**) abdominal fat coefficient. Results are presented as mean ± standard deviation (*n* = 5). Different lowercase letters above the bars indicate significant differences between groups (*p* < 0.05).

As shown in Figure 3, different dietary fat interventions exerted distinct effects on organ coefficients in mice. No significant differences in heart coefficient were observed across groups (*p* > 0.05). Compared with the CK group, liver coefficients in the RO and LSO groups remained unchanged, whereas those in the CA and HSO groups were significantly increased (*p* < 0.05). Additionally, the CA and HSO groups exhibited significantly lower kidney and brain coefficients, but markedly higher perirenal and abdominal fat indices (*p* < 0.05). In contrast, no significant changes in these organ coefficients were detected in the RO and LSO groups relative to the control.

### 3.4. Serum Lipid Profiles

**Figure 4:**
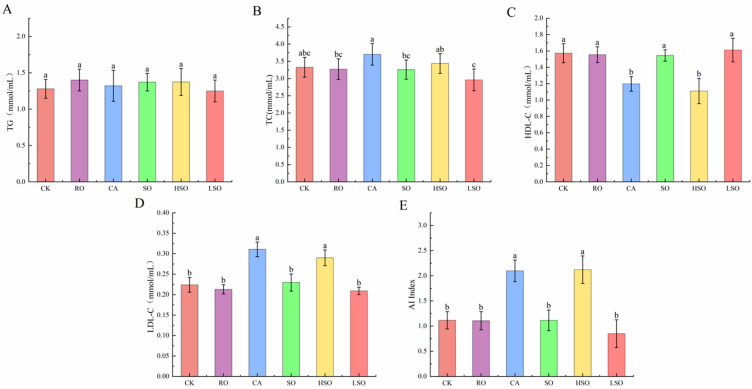
Serum lipid profiles in mice: (**A**) TG content, (**B**) TC content, (**C**) HDL-C content, (**D**) LDL-C content, and (**E**) AI. Results are presented as mean ± standard deviation (*n* = 5). Different lowercase letters above the bars indicate significant differences between groups (*p* < 0.05).

The effects of different oil intake on serum lipid levels in mice are shown in Figure 4. No significant difference was observed in triglyceride (TG) levels among the groups (ANOVA: F = 0.671, *p* = 0.649, η^2^ = 0.123, medium effect size). In contrast, total cholesterol (TC) levels differed significantly (ANOVA: F = 3.359, *p* = 0.019, η^2^ = 0.412, large effect size). The CA group showed significantly higher TC (3.70 mmol/L) than the RO, SO, and LSO groups (*p* < 0.05), while the LSO group had significantly lower TC (2.96 mmol/L) than the CA and HSO groups (*p* < 0.05). Both HDL-C and LDL-C levels exhibited highly significant differences among groups (ANOVA: F = 17.774, *p* < 0.001, η^2^ = 0.787; F = 33.544, *p* < 0.001, η^2^ = 0.875; both large effect sizes). The CA and HSO groups had significantly lower HDL-C levels (1.20 and 1.11 mmol/L, respectively) than the CK, RO, SO, and LSO groups (*p* < 0.05), and significantly higher LDL-C levels than the same groups (*p* < 0.05). The atherogenic index (AI) also differed significantly (ANOVA: F = 31.165, *p* < 0.001, η^2^ = 0.866, large effect size), with the CA and HSO groups showing significantly higher AI values than all other groups (*p* < 0.05). Detailed ANOVA results are provided in Supplementary Table S4. These findings indicate that intake of CA and HSO induced dyslipidemia in mice, potentially increasing the risk of lipid metabolism-related disorders. In contrast, the RO and LSO groups exhibited relatively stable lipid profiles, with the LSO group showing the lowest TC and LDL-C levels, suggesting a potential lipid-lowering benefit. Overall, the results demonstrate that different types of dietary oils have distinct effects on serum lipid metabolism in mice.

### 3.5. Histopathological Evaluation of the Colon

**Figure 5:**
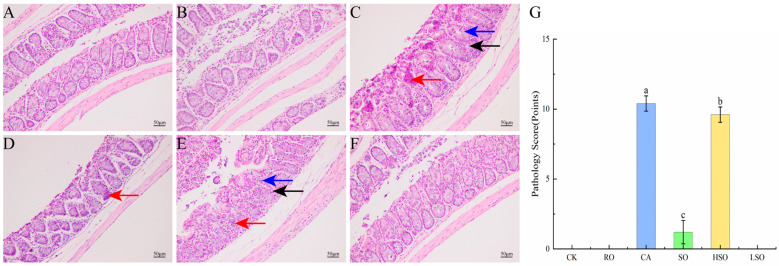
Pathological observation of the colon (scale bar, 50 µm). (**A**) CK group, (**B**) RO group, (**C**) CA group, (**D**) SO group, (**E**) HSO group, (**F**) LSO group, and (**G**) Histological score. Results are presented as mean ± standard deviation (*n* = 5). Different lowercase letters above the bars indicate significant differences between groups (*p* < 0.05).

As shown in Figure 5, compared with the control group (CK), the RO and LSO groups exhibited normal colonic morphology, characterized by well-arranged glands, intact mucosa, relatively preserved goblet cells and crypts, and absence of significant inflammation or necrosis. In contrast, the CA and HSO groups showed severe inflammatory cell infiltration (red arrows), disrupted goblet cells (blue arrows), and damaged crypts (black arrows). The SO group also displayed notable inflammatory cell infiltration (red arrows), indicating apparent inflammation in the CA, SO, and HSO groups. Histopathological scoring revealed significantly higher scores in the CA, SO, and HSO groups compared to CK (*p* < 0.05), with the severity ranking as CA > HSO > SO. These results demonstrate that intake of RO and LSO did not induce organic pathological changes in the colon, whereas consumption of CA, SO, and HSO triggered inflammatory responses.

### 3.6. qPCR Results

**Figure 6:**
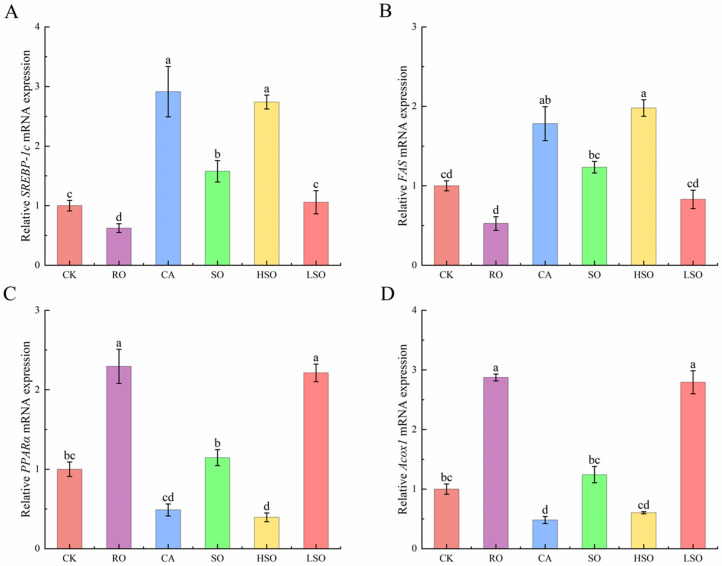
Expression of lipid metabolism-related genes. (**A**) Relative *SREBP-1c* mRNA expression, (**B**) Relative *FAS* mRNA expression, (**C**) Relative *PPARα* mRNA expression, (**D**) Relative *Acox1* mRNA expression. Results are presented as mean ± standard deviation (*n* = 5). Different lowercase letters above the bars indicate significant differences between groups (*p* < 0.05).

To further investigate the regulatory effects of different dietary oils on lipid metabolism in mice, we quantified the mRNA expression levels of key genes involved in fatty acid synthesis and oxidation using qPCR. As shown in Figure 6, compared with the CK group, the expression of *SREBP-1c* was significantly up-regulated in the CA, SO, and HSO groups, while *FAS* expression was markedly increased in the CA and HSO groups. In contrast, the expression of *PPARα* and *Acox1* was significantly elevated in the RO and LSO groups (*p* < 0.05).

**Figure 7:**
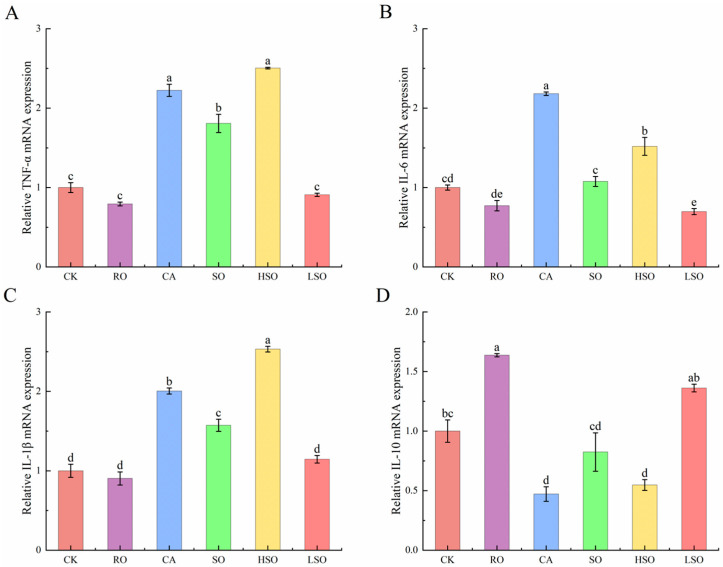
Expression of inflammation factor-related genes. (**A**) Relative TNF-α mRNA expression, (**B**) Relative IL-6 mRNA expression, (**C**) Relative IL-1β mRNA expression, (**D**) Relative IL-10 mRNA expression. Results are presented as mean ± standard deviation (*n* = 5). Different lowercase letters above the bars indicate significant differences between groups (*p* < 0.05).

As shown in Figure 7, compared with the CK group, the expression levels of TNF-α were significantly up-regulated in the CA, SO, and HSO groups; IL-6 expression was markedly increased in the CA and HSO groups; and Interleukin-1 beta (IL-1β) levels were significantly elevated in the CA, SO, and HSO groups (*p* < 0.05). In contrast, the RO and LSO groups showed up-regulated expression of Interleukin-10 (IL-10).

### 3.7. Gut Microbiota Composition Analysis

**Figure 8:**
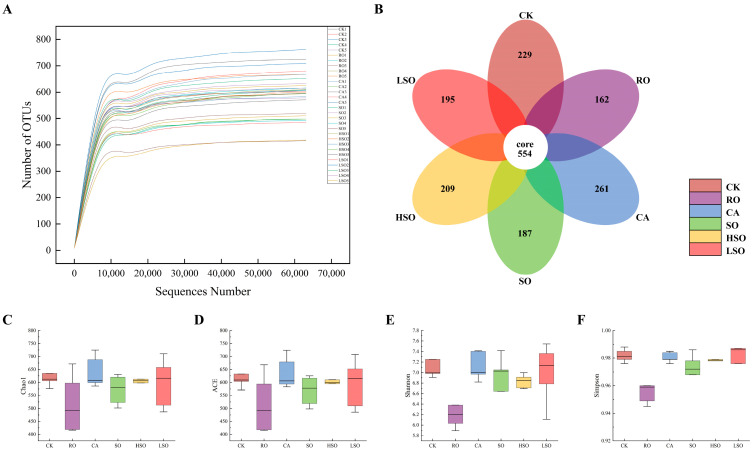
Intestinal microbial diversity: (**A**) sample dilution curves, (**B**) OTU petal plots, (**C**) Chao1 index, (**D**) ACE index, (**E**) Shannon index, and (**F**) Simpson index.

To investigate the impact of different oil diets on gut microbiota, cecal microbial composition was analyzed via 16S rRNA gene sequencing. The dilution curve (Figure 8A) reflects sequencing depth; its plateauing slope indicates sufficient coverage and reliable data quality. OTU analysis revealed 554 shared OTUs across all groups, with unique OTUs in the CK, RO, CA, SO, HSO, and LSO groups numbering 229, 162, 261, 187, 209, and 195, respectively (Figure 8B). α-diversity was assessed using Chao1, ACE (richness indicators), Shannon, and Simpson (diversity indicators). Higher Chao1 and ACE values reflect greater species richness, while higher Shannon values indicate higher diversity; conversely, higher Simpson values indicate lower diversity. As shown in Figure 8C–F, the CA group exhibited the highest Chao1 and ACE indices. Compared to the CK group, no significant differences in α-diversity indices were found in the CA, SO, HSO, or LSO groups, whereas the RO group showed significantly lower values across all indices. This suggests that CA, SO, HSO, and LSO do not substantially alter microbial richness or evenness, while RO intake reduces both abundance and diversity of the gut microbiota.

**Figure 9:**
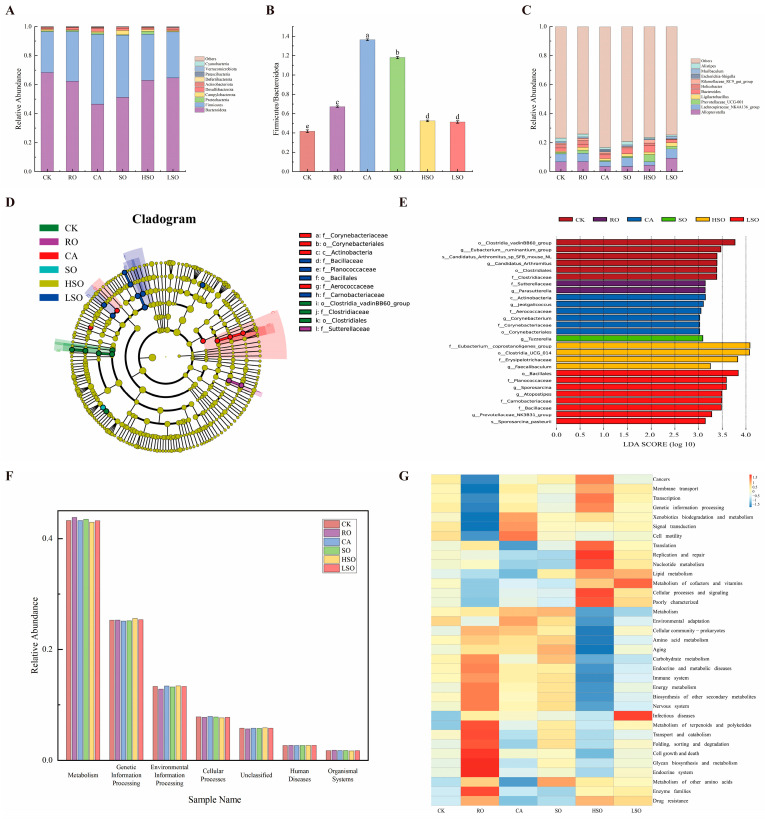
Gut microbiota analysis: (**A**) relative abundance at the phylum level, (**B**) relative abundance at the genus level, and (**C**) species abundance stacked bar chart; LEfSe analysis results: (**D**) phylogenetic tree and (**E**) LDA score distribution histogram; functional prediction analyses: (**F**) stacked bar chart and (**G**) heatmap.

At the phylum level (Figure 9A), six taxa exceeded 1% relative abundance in all groups: *Bacteroidota*, *Firmicutes*, *Proteobacteria*, *Campylobacterota*, *Desulfobacterota*, and *Actinobacteriota*. *Firmicutes* and *Bacteroidota* were dominant, confirming their central role in murine gut ecology. *Bacteroidota* abundance was highest in the CK group, intermediate in RO, HSO, and LSO, and lowest in CA; conversely, *Firmicutes* abundance followed the inverse pattern, peaking in CA. *Proteobacteria* was most abundant in CA and least in LSO; *Campylobacterota* peaked in SO; *Desulfobacterota* was highest in CA; and *Actinobacteriota* reached maximum levels in LSO. Furthermore, the Firmicutes/Bacteroidota (F/B) ratio increased across oil-fed groups compared with CK, with the highest ratio observed in the CA group (Figure 9B), indicating a shift toward a microbiota profile associated with metabolic dysfunction.

At the genus level (Figure 9C), *Alloprevotella* abundance followed the order LSO > RO > CK > HSO > CA > SO. *Lachnospiraceae_NK4A136_group* was most abundant in LSO and lowest in HSO. *Prevotellaceae_UCG-001* was enriched in HSO and moderately present in LSO and CK. *Ligilactobacillus* was most prevalent in LSO. *Bacteroides* abundance decreased in RO and LSO but increased in CA, SO, and HSO. *Helicobacter* was lowest in the LSO group.

Linear discriminant analysis Effect Size (LEfSe) analysis included phylogenetic discriminant analysis (Figure 9D) and linear discriminant analysis (LDA) score bar plots (Figure 9E, threshold LDA > 3). At the class level, *Actinobacteria* was enriched in CA. At the order level, *Clostridia*_vadinBB60 and *Clostridiales* were enriched in CK; *Orynebacteriales* in CA; *Clostridia* UCG-014 in HSO; and *Bacillales* in LSO. At the family/genus level, *Clostridiaceae* was enriched in CK; *Sutterellaceae* in RO; *Aerococcaceae* and *Corynebacteriaceae* in CA; *Erysipelotrichaceae* in HSO; and *Planococcaceae*, *Carnobacteriaceae*, and *Bacillaceae* in LSO. To evaluate the overall impact of different dietary oils on the gut microbiota structure in mice, we performed ADONIS (PERMANOVA) analysis. The results demonstrated a significant alteration in the microbial community structure following oil administration (*p* < 0.05 or *p* < 0.01). Detailed ADONIS statistical results are provided in Supplementary Table S5.

Functional prediction based on Kyoto Encyclopedia of Genes and Genomes (KEGG) annotation (Figure 9F) revealed that “Metabolism” was the most abundant first-level pathway, followed by “Genetic Information Processing” and “Environmental Information Processing,” aligning with core microbial functions in energy maintenance and environmental adaptation. Differential analysis of secondary pathways (Figure 9G) showed that RO and HSO had the most pronounced effects. In the RO group, pathways related to cell growth and death, glycan biosynthesis and metabolism, and the endocrine system were significantly upregulated, while membrane transport, xenobiotic biodegradation and metabolism, and signal transduction were significantly downregulated. In the HSO group, replication and repair, nucleotide metabolism, lipid metabolism, and cell processes and signaling were significantly upregulated, whereas cell community, amino acid metabolism, aging, and carbohydrate metabolism were significantly downregulated. In the CA group, xenobiotic biodegradation and metabolism, signal transduction, cell motility, and amino acid metabolism were significantly upregulated, while translation, other amino acid metabolism, and drug resistance were significantly downregulated. In the SO group, metabolism, amino acid metabolism, aging, and carbohydrate metabolism were significantly upregulated, whereas replication and repair and nucleotide metabolism were significantly downregulated. The LSO group exerted minimal overall impact, with upregulation in infectious disease metabolism and cofactor and vitamin metabolism pathways, and downregulation in general metabolism. It is speculated that obesity in the CA and HSO groups may result from upregulated lipid and amino acid metabolism promoting fat synthesis, while colonic inflammation could be linked to impaired carbohydrate and amino acid metabolic pathways. The aforementioned predictive analysis based on the KEGG database revealed that the obesity phenotype observed in the CA and HSO groups may be associated with predicted up-regulation of pathways such as lipid metabolism and signal transduction, while their colonic inflammation might be linked to predicted down-regulation of pathways including carbohydrate metabolism. These functional predictions provide valuable clues for elucidating the underlying mechanisms, though further experimental validation is required.

### 3.8. SCFAs Analysis

**Figure 10:**
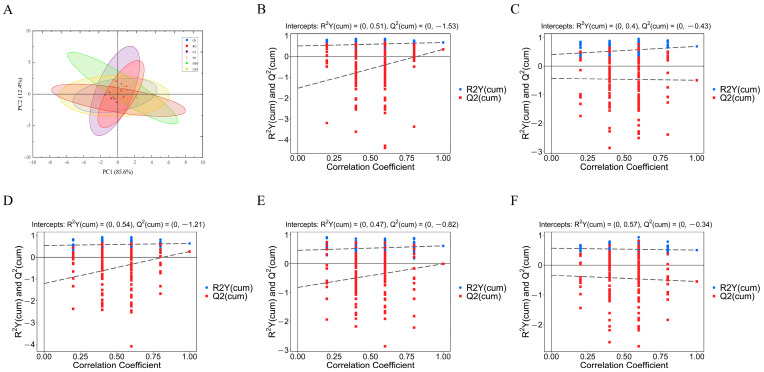
(**A**) PCA; (**B**) comparison between CK group and RO group; (**C**) comparison between CK group and CA group; (**D**) comparison between CK group and SO group; (**E**) comparison between CK group and HSO group; (**F**) comparison between CK group and LSO group.

Principal component analysis (PCA) is an unsupervised pattern recognition method used for statistical analysis of multivariate data, enabling initial assessment of metabolic differences between samples and intra-group variation. As shown in Figure 10A, the PCA score plot (PC1: 85.6%; PC2: 12.4%) reveals a clear separation trend among the RO, CA, SO, HSO, and CK groups, with particularly distinct clustering observed among the CA, HSO, and CK groups. In contrast, no obvious separation is evident between the LSO and CK groups. This indicates significant alterations in cecal SCFAs profiles following consumption of different oils, with the most pronounced metabolic divergence observed in mice fed CA and HSO, likely attributable to their higher SFA and lower UFA content. Meanwhile, samples within each group are tightly clustered, reflecting low intra-group variability and high experimental reproducibility.

To further investigate intergroup metabolite differences, orthogonal partial least squares discriminant analysis (OPLS-DA) was employed to identify differential variables and calculate variable importance in projection (VIP) values. R^2^Y and Q^2^ values approaching 1 indicate robust model fitting and predictive capability. Additionally, the OPLS-DA models were validated using 200 permutation tests to rule out overfitting. Clear group separations were observed in both positive and negative ion modes, confirming significant metabolic distinctions between dietary groups.

In Figure 10B–F, the x-axis represents the permutation retention degree (i.e., the proportion of permutations where the order of the Y-variable matches that of the original model; the point at a permutation retention degree of 1 corresponds to the R^2^Y and Q^2^ values of the original model). The y-axis represents the values of R^2^Y and Q^2^. The blue dots indicate the R^2^Y values obtained from the permutation test, while the red squares indicate the Q^2^ values obtained from the test. The two dashed lines represent the regression lines for R^2^Y and Q^2^, respectively.

**Figure 11:**
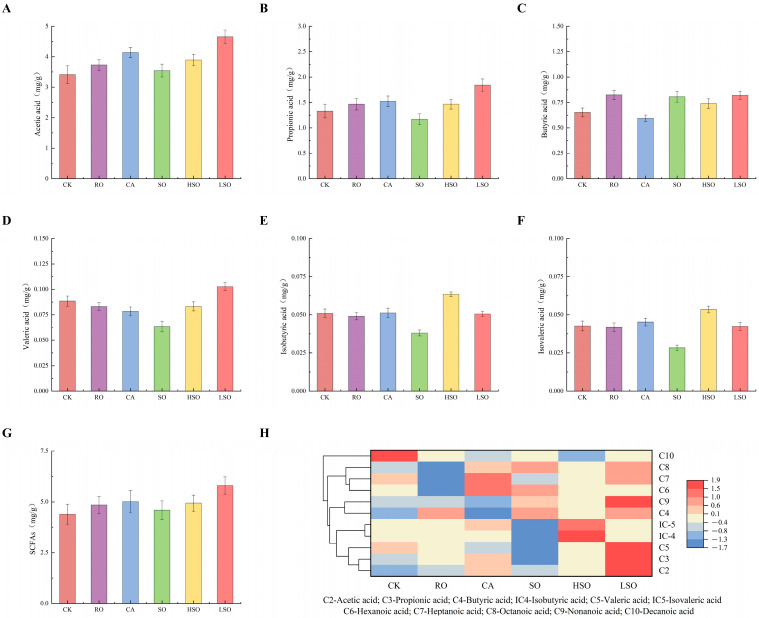
Short-chain fatty acid contents: (**A**) acetic acid, (**B**) propionic acid, (**C**) butyric acid, (**D**) valeric acid, (**E**) isobutyric acid, (**F**) isovaleric acid, (**G**) total short-chain fatty acids, and (**H**) cluster heatmap.

Figure 11 presents the relative abundance changes in various SCFAs in the cecum of mice after the intake of different oils. The heatmap color scale ranges from blue (low) to red (high), representing increasing metabolite levels. Metabolic profiles of the CK and RO groups: SCFAs levels in these groups are generally moderate, without marked up- or down-regulation of specific metabolites. Metabolic profiles of the CA and SO groups: compared to CK, the CA group exhibits elevated levels of hexanoic and heptanoic acids, along with significantly reduced butyric acid. In contrast, the SO group shows significantly lower acetic and propionic acid levels but markedly increased butyric acid. Metabolic profile of the HSO group: this group displays significantly higher relative abundances of isovaleric and isobutyric acids compared to other groups. Metabolic profile of the LSO group: LSO-fed mice exhibit relatively high levels across multiple SCFAs. Specifically, acetic, propionic, and butyric acid levels are all elevated compared to those in the CK group. Overall, heatmap clustering demonstrates that different oil intakes distinctly modulate SCFAs metabolism, leading to divergent metabolic signatures among groups. Notably, the LSO group promotes the production of beneficial SCFAs such as butyric and propionic acids, suggesting a potential regulatory effect on intestinal health. Of note, the LSO group exhibited higher relative levels of beneficial short-chain fatty acids, such as butyrate and propionate, which aligns with the healthy histological profile observed in the colon of this group.

KEGG pathway enrichment analysis revealed that Altay sheep rump fat and its fractionated products significantly influenced carbohydrate metabolism, digestive system function, and lipid metabolism in mice. In carbohydrate metabolism pathways, no significant differences in propionate or butyrate metabolism regulation were observed among groups (*p* > 0.05). However, compared to CK, the RO, CA, SO, and HSO groups exhibited significant upregulation of glycolysis/gluconeogenesis and pyruvate metabolism (*p* < 0.05), whereas the LSO group showed significant downregulation in these pathways (*p* < 0.05). Regarding digestive system pathways, protein digestion and absorption as well as carbohydrate digestion and absorption were significantly enhanced in the RO, CA, and HSO groups (*p* < 0.001), while protein digestion and absorption were significantly reduced in the SO and LSO groups (*p* < 0.001), despite increased carbohydrate digestion and absorption (*p* < 0.001). For energy metabolism and cofactor/vitamin metabolism pathways, no significant intergroup differences were detected (*p* > 0.05). In lipid metabolism, the RO and LSO groups showed significant downregulation compared to CK (*p* < 0.01). Significant upregulation was observed in pathways for endocrine and metabolic diseases related to glycan biosynthesis/metabolism, as well as in nervous system pathways such as taurine/hypotaurine metabolism, in the RO, CA, SO, and HSO groups. In contrast, the LSO group showed significant downregulation in these pathways, as shown in Supplementary Figure S1.

## 4. Discussion

This study demonstrates that solvent fractionation of Altay sheep rump fat yields two distinct fractions HSO and LSO—which exert divergent physiological effects in mice. Specifically, dietary intake of HSO and CA induced obesity, dyslipidemia, and colonic inflammation, whereas no significant adverse effects were observed in mice fed LSO or RO, with LSO showing a notable advantage in promoting intestinal health. Beyond systematically characterizing these phenotypic outcomes, this study integrates gut microbiota and metabolite profiling to elucidate how different lipid components modulate host lipid metabolism through regulation of the intestinal microecology. Based on the findings of this study, we propose a potential pathway that can be summarized as follows: the fatty acid composition of dietary lipids may drive structural and functional differentiation of the gut microbiota, which in turn influences the profile of microbial metabolites. These changes may collectively mediate the different physiological phenotypes observed in the host.

Physicochemical characterization revealed that CA and HSO had high SFA content, while RO and LSO were enriched in UFAs. These findings align with those of Wang et al. [27] and Pădureţ [28]. The physicochemical properties of oils are closely linked to their fatty acid composition [29]. The high melting points of CA and HSO are attributable to their elevated SFAs levels, whereas the high iodine value and low melting point of RO result from its high UFAs content [30]. Notably, fractionation effectively modified the lipid profile of sheep rump fat by enriching SFAs in HSO and significantly increasing UFAs in LSO. More importantly, compared with SO, both the acid value and peroxide value were significantly increased in HSO but significantly decreased in LSO, indicating that the fractionation process can reduce the lipid oxidation risk in the low-melting-point fraction [31]. This improvement may be attributed to the enrichment of antioxidants in the liquid oil during fractionation, combined with the effect of the lower fractionation temperature [32,33].

The core finding of this study is that intake of different oils is significantly associated with specific alterations in host metabolic phenotypes and gut microbiota. Specifically, consumption of CA and HSO was linked to increased obesity indices, dyslipidemia, and colonic inflammation in mice. At the molecular level, mRNA expression of key lipogenic genes *SREBP-1c* and *FAS* was significantly up-regulated in the livers of the CA and HSO groups, whereas key fatty acid oxidation genes *PPARα* and *Acox1* were markedly up-regulated in the RO and LSO groups. This suggests that HSO and CA may promote lipid accumulation by enhancing hepatic lipogenesis and suppressing oxidative breakdown, while LSO likely activates fatty acid oxidation pathways, facilitating lipid catabolism—consistent with its stable blood lipid profile and lower obesity indices. Notably, these gene expression changes may not be isolated events but are interrelated with structural and functional shifts in the gut microbiota. The underlying mechanism may involve a cascade mediated by gut microbiota and their metabolites. First, the high SFA content in CA and HSO was associated with gut microbiota dysbiosis, characterized by an increased relative abundance of pro-inflammatory *Desulfovibrio* and a decrease in beneficial *Bacteroidetes*—a microbial signature consistent with obesity and related metabolic disorders [34,35,36]. Second, KEGG pathway prediction based on 16S rRNA gene sequencing indicated up-regulation of lipid synthesis and storage-related pathways in the CA and HSO groups [37], suggesting that microbial metabolic shifts may create an environment conducive to host fat accumulation. It should be emphasized that such functional predictions are inferential and require validation via metagenomic or metabolomic approaches. Simultaneously, SCFAs profiling provided direct metabolic evidence: butyrate levels were significantly reduced in the CA group, whereas branched-chain fatty acids (BCFAs) such as isobutyrate and isovalerate were elevated in the HSO group. Reduced butyrate is associated with impaired barrier function and anti-inflammatory capacity [38], while elevated BCFAs often indicate protein fermentation, potentially accompanied by harmful metabolites like hydrogen sulfide [39,40]. These SCFAs alterations correspond with the inflammatory state in colon tissue: pro-inflammatory cytokines TNF-α, IL-6, and IL-1β were significantly up-regulated in CA and HSO groups, whereas the anti-inflammatory cytokine IL-10 was higher in RO and LSO groups. This clearly indicates that CA and HSO intake is associated with significant colonic inflammation, while LSO exhibits anti-inflammatory properties—consistent with histopathological observations. Furthermore, microbiota dysbiosis and associated metabolite changes may indirectly affect lipid metabolism—for example, by reducing HDL-C synthesis and promoting LDL-C production [41]—aligning with our experimental results. In contrast, mice fed RO and LSO showed no significant differences from the CK group in obesity indices, blood lipid levels, or colonic health. Notably, LSO, as a modified animal fat with a healthier UFA-rich profile achieved through fractionation, was associated with a more favorable gut microecological environment. The microbial structure in the LSO group resembled that of the CK group; importantly, LSO promoted beneficial SCFAs (e.g., butyrate, propionate, acetate), which help energize colonocytes and strengthen barrier function [42,43]—likely a key mechanism maintaining colonic health.

Although HSO and LSO showed similar relative abundances at the phylum level (e.g., *Firmicutes*, *Bacteroidetes*) and F/B ratios, significant differences emerged at finer taxonomic levels (e.g., family, genus), which may explain their divergent metabolic phenotypes. This suggests that phylum-level ratios may be insufficient to fully reveal microbial function [44], and that specific key taxa and their functional capacities drive phenotypic differences. We found that at the genus level, the LSO group had higher relative abundances of beneficial bacteria such as *Ligilactobacillus* and *Lachnospiraceae_NK4A136_group*—the latter reported to promote SCFAs production and enhance the gut barrier [45]. In contrast, the HSO group showed a higher relative abundance of *Erysipelotrichaceae*, a family positively correlated with obesity and inflammation [46]. These results indicate that high-SFA oil (e.g., HSO) intake is associated with proliferation of pro-inflammatory and potentially pathogenic bacteria, while high-UFA oil (e.g., LSO) intake is linked to maintenance of beneficial taxa—consistent with recent studies suggesting that specific genera better reflect dietary fat-health relationships [47]. Integrating microbiota structure, SCFAs profiles, and host gene expression data, we propose that LSO may improve lipid metabolism and gut health through a potential synergistic pathway involving enrichment of specific beneficial bacteria, promotion of beneficial SCFAs production, and up-regulation of fatty acid oxidation genes.

This study has several limitations. Although gavage ensured precise dosing, digestive and absorptive processes may differ from spontaneous dietary intake. The absence of pair-feeding to control total energy intake means that observed phenotypes result from combined effects of fat type and potential differences in food consumption. The 40-day experimental period and limited sample size may be insufficient to reveal long-term metabolic adaptations or subtle effects. The exclusive use of male mice warrants caution in generalizing results. Future research should adopt isocaloric feeding models, extend intervention periods, integrate multi-omics with in vitro studies, and include human trials to more systematically elucidate the mechanisms and potential applications of different sheep rump fat fractions on health.

## 5. Conclusions

This study demonstrates that solvent fractionation of sheep rump fat yields low-melting-point liquid oil (LSO) and high-melting-point solid fat (HSO) with distinct effects on lipid metabolism and gut health in mice. LSO intake maintained relatively stable blood lipid levels and healthy colonic morphology, whereas HSO showed opposing effects. These findings indicate that solvent fractionation can directionally modify the fatty acid composition of sheep rump fat, thereby potentially influencing its metabolic effects in vivo. This work provides preliminary experimental evidence for further investigation into the health impacts of different animal fat components.

## Figures and Tables

**Table 1:** Nutritional components of mice diet.

Parameter	Content	Parameter	Content
Calories	3766 KJ/Kg	Ash	4.17%
Calories from Protein	19.3%	Calcium (Ca)	0.046%
Calories from Fat	16.7%	Total Phosphorus (P)	0.036%
Moisture	6.6%	Ca:P Ratio	1.28
Crude Fat	7.0%	Lysine	0.028%
Carbohydrates	64.3%	Methionine + Cystine	0.017%

**Table 2:** Main fatty acids composition.

Fatty Acid	RO	CA	SO	HSO	LSO
C14:0	-	2.35 ± 0 ^d^	7.35 ± 0.02 ^b^	10.17 ± 0.01 ^a^	5.2 ± 0.02 ^c^
C16:0	3.98 ± 0 ^e^	43.75 ± 0.01 ^a^	25.19 ± 0.01 ^c^	33.4 ± 0.02 ^b^	21.62 ± 0.09 ^d^
C16:1	-	1.11 ± 0 ^d^	3.1 ± 0.01 ^b^	2.14 ± 0 ^c^	3.45 ± 0.01 ^a^
C18:0	2.03 ± 0.03 ^e^	19.44 ± 0.01 ^a^	11.38 ± 0.02 ^c^	17.2 ± 0.01 ^b^	9.23 ± 0.04 ^d^
C18:1n-9t	-	1.59 ± 0.02 ^c^	4.62 ± 0 ^ab^	4.52 ± 0.01 ^b^	4.83 ± 0.21 ^a^
C18:1n-9c	59.64 ± 0.13 ^a^	26.25 ± 0 ^d^	35.61 ± 0.01 ^c^	22.5 ± 0.02 ^e^	41.83 ± 0 ^b^
C18:2n-6t	3.14 ± 0.01 ^a^	0.55 ± 0 ^e^	0.84 ± 0 ^c^	0.59 ± 0 ^d^	0.95 ± 0.01 ^b^
C18:2n-6c	19.56 ± 0.09 ^a^	3.56 ± 0 ^b^	2.56 ± 0 ^d^	1.44 ± 0 ^e^	3 ± 0.01 ^c^
C18:3n-3c	6.07 ± 0.04 ^a^	-	1.94 ± 0.01 ^c^	1.08 ± 0 ^d^	2.26 ± 0.01 ^b^
SFA	6.01 ± 0.03 ^e^	65.54 ± 0.02 ^a^	43.91 ± 0.01 ^c^	60.77 ± 0.03 ^b^	36.05 ± 0.15 ^d^
UFA	88.41 ± 0.05 ^a^	33.06 ± 0.01 ^d^	48.67 ± 0.01 ^c^	32.27 ± 0.03 ^e^	56.32 ± 0.18 ^b^
MUFA	59.64 ± 0.13 ^a^	27.36 ± 0 ^d^	38.7 ± 0.01 ^c^	24.65 ± 0.02 ^e^	45.28 ± 0.01 ^b^
PUFA	28.77 ± 0.11 ^a^	5.7 ± 0.01 ^e^	9.97 ± 0.01 ^c^	7.63 ± 0.01 ^d^	11.04 ± 0.19 ^b^

Results are presented as mean ± standard deviation (*n* = 3). Different lowercase letters in the table indicate significant differences between groups (*p* < 0.05); ‘-’ means not detected.

## Data Availability

The original contributions presented in the study are included in the article, further inquiries can be directed to the corresponding author.

## Supplementary Materials

The following supporting information can be downloaded at: https://www.mdpi.com/article/10.3390/foods14213641/s1, Figure S1: KEGG pathway enrichment differential analysis: (A) CK group versus RO group, (B) CK group versus CA group, (C) CK group versus SO group, (D) CK group versus HSO group, and (E) CK group versus LSO group (* *p* < 0.05, ** *p* < 0.01, *** *p* < 0.001).; Table S1: Histological Injury Scoring Criteria for Colon Tissue; Table S2. The primer sequences for real-time quantitative PCR; Table S3. ANOVA of Growth Indicators in Mice Fed Different Oils; Table S4. ANOVA of Serum Lipid Levels in Mice Fed Different Oils; Table S5. ADONIS (PERMANOVA) Statistical Test Results.
