# Supplementary material for: Solvent Fractionation Improves the Functional Properties of Sheep Rump Fat: Effects of Different Lipid Fractions on Lipid Metabolism and Gut Health in Mice
Source: Foods. 2025 Oct 24;14(21):3641. doi: 10.3390/foods14213641 (PMC12607981; doi:10.3390/foods14213641)
Supplement: Supplementary file 1 [file foods-14-03641-s001.zip › foods-3913821-supplementary.pdf]

Table S1. Histological Injury Scoring Criteria for Colon Tissue

Score	Inflammation Severity	Lesion Depth	Crypt Damage	Affected Area (%)
0	None	None	None	None
1	Mild	Submucosa	1/4 crypts damaged	1–25
2	Severe	Muscularis	1/2 crypts damaged	26–50
3		Serosa	3/4 crypts damaged	51–75
4			Complete crypt loss	76–100

Table S2. The primer sequences for real-time quantitative PCR.

Gene	Product size (bp)	Primer pair	Primer Sequence (5′-3′)
β-actin	80	Forward	ACTGCCGCATCCTCTTCCTC
		Reverse	AACCGCTCGTTGCCAATAGTG
GAPDH	80	Forward	GCAAATTCAACGGCACAGTCAAG
		Reverse	TCGCTCCTGGAAGATGGTGATG
FAS	101	Forward	TCCTGAAGCCGAACACCTCTG
		Reverse	GCGACAATATCCACTCCCTGAATC
Acox1	104	Forward	GACCTGAGTGAGCTGCCTGAG
		Reverse	CCGCAAGCCATCCGACATTC
PPAR*α*	138	Forward	CGGGAAAGACCAGCAACAACC
		Reverse	AGCAGTGGAAGAATCGGACCTC
SREBP-1c	136	Forward	CGCTACCGTTCCTCTATCAATGAC
		Reverse	TCTGGTTGCTGTGCTGTAAGAAG
IL-6	102	Forward	CGGAGAGGAGACTTCACAGAGG
		Reverse	TTCCACGATTTCCCAGAGAACATG
IL-10	81	Forward	GGACAACATACTGCTAACCGACTC
		Reverse	TGGATCATTTCCGATAAGGCTTGG
IL-1β	94	Forward	TCGCAGCAGCACATCAACAAG
		Reverse	TCCACGGGAAAGACACAGGTAG
TNF-*α*	86	Forward	ACGTGGAACTGGCAGAAGAGG
		Reverse	TGAGAAGAGGCTGAGACATAGGC

Table S3. ANOVA of Growth Indicators in Mice Fed Different Oils

Indicator	Source of Variation	Sum of Squares	df	Mean Square	F-value	p-value	η^2
Body Weight	Between Groups	107.717	5	21.543	5.643	0.001	0.540
	Within Groups	91.618	24	3.817			
	Total	199.335	29				
Body Length	Between Groups	0.791	5	0.158	1.357	0.275	0.220
	Within Groups	2.796	24	0.117			
	Total	3.587	29				
BMI	Between Groups	0.003	5	0.001	3.218	0.023	0.401

	Within Groups	0.005	24	0.000			
	Total	0.008	29				
Lee's	Between Groups	612.663	5	122.533	3.594	0.014	0.480
	Within Groups	818.190	24	34.091			
	Total	1430.853	29				

Table S4. ANOVA of Serum Lipid Levels in Mice Fed Different Oils

Indicator	Source of Variation	Sum of Squares	df	Mean Square	F-value	p-value	η^2
TG	Between Groups	0.087	5	0.018	0.671	0.649	0.123
	Within Groups	0.624	24	0.026			
	Total	0.712	29				
TC	Between Groups	1.479	5	0.296	3.359	0.019	0.412
	Within Groups	2.113	24	0.088			
	Total	3.592	29				
HDL-C	Between Groups	1.190	5	0.238	17.774	0.000	0.787
	Within Groups	0.321	24	0.013			
	Total	1.511	29				
LDL-C	Between Groups	0.047	5	0.009	33.544	0.000	0.875
	Within Groups	0.007	24	0.000			
	Total	0.053	29				
AI	Between Groups	7.785	5	1.557	31.165	0.000	0.866
	Within Groups	1.199	24	0.050			
	Total	8.984	29				

Table S5. ADONIS (PERMANOVA) Statistical Test Results

Group Comparison	df	Sum of Squares	Mean Square	F-value	R^2	p-value
CK-RO	1	0.36531	0.36531	1.97809	0.19824	0.012
CK-CA	1	0.28866	0.28866	1.55507	0.16275	0.012
CK-SO	1	0.26532	0.26532	1.4297	0.15162	0.045
CK-HSO	1	0.2863	0.2863	1.90876	0.19263	0.009
CK-LSO	1	0.41083	0.41083	2.33104	0.22563	0.010

A

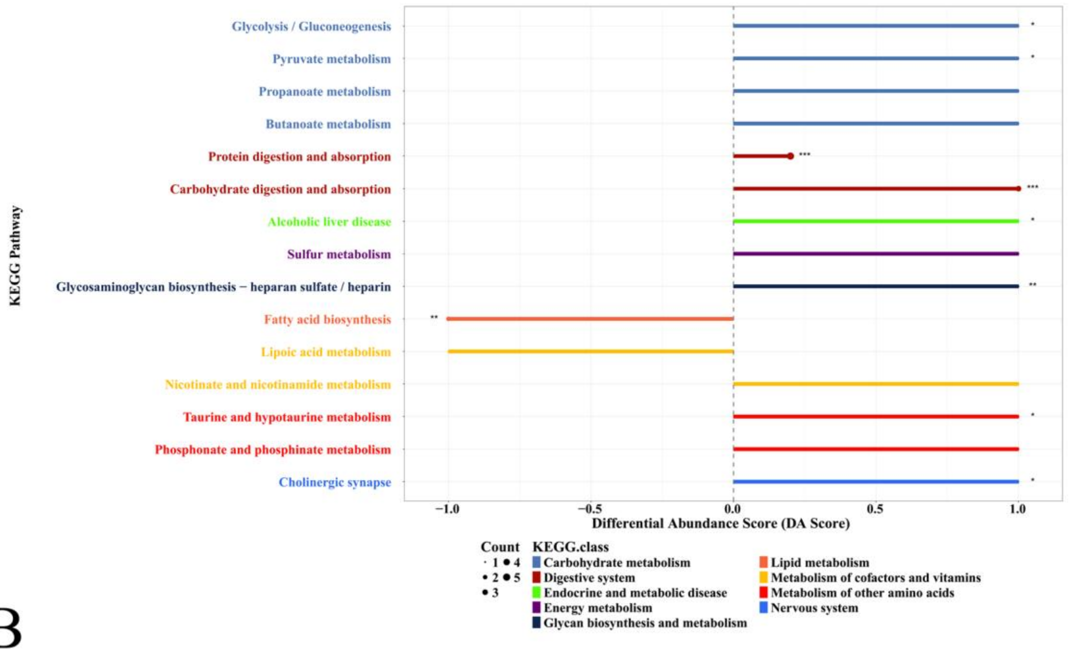
KEGG Pathway
Glycolysis / Gluconeogenesis
Pyruvate metabolism
Propanoate metabolism
Butanoate metabolism
Protein digestion and absorption
Carbohydrate digestion and absorption
Alcoholic liver disease
Sulfur metabolism
Glycosaminoglycan biosynthesis − heparan sulfate / heparin
Fatty acid biosynthesis
Lipoic acid metabolism
Nicotinate and nicotinamide metabolism
Taurine and hypotaurine metabolism
Phosphonate and phosphinate metabolism
Cholinergic synapse
−1.0
−0.5
0.0
0.5
1.0
Differential Abundance Score (DA Score)
Count
1
2
3
4
5
KEGG.class
Carbohydrate metabolism
Digestive system
Endocrine and metabolic disease
Energy metabolism
Glycan biosynthesis and metabolism
Lipid metabolism
Metabolism of cofactors and vitamins
Metabolism of other amino acids
Nervous system

B

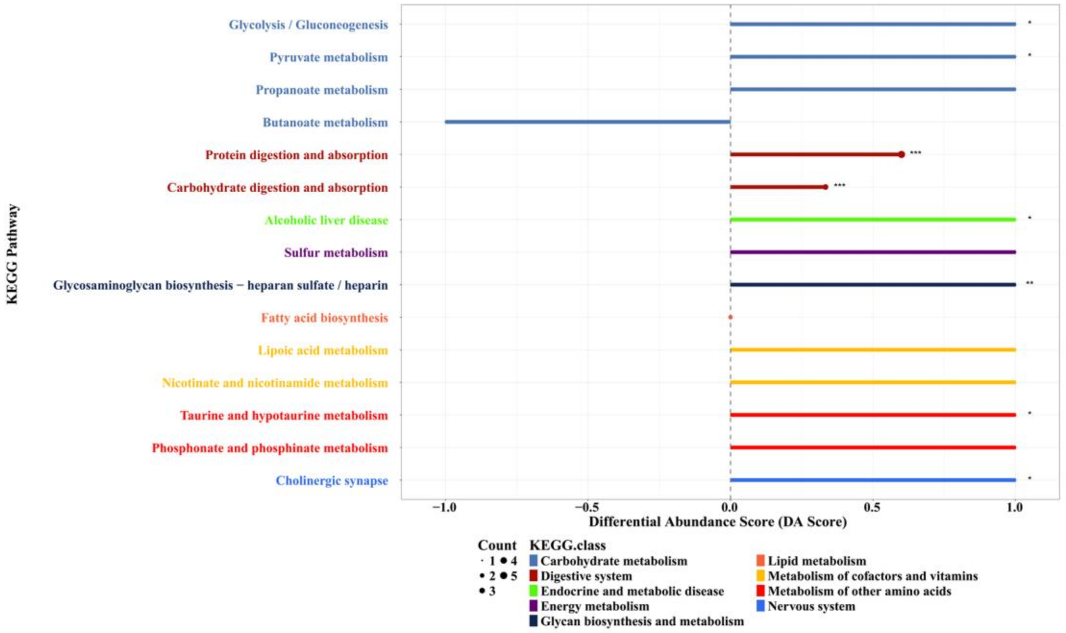
KEGG Pathway
Glycolysis / Gluconeogenesis
Pyruvate metabolism
Propanoate metabolism
Butanoate metabolism
Protein digestion and absorption
Carbohydrate digestion and absorption
Alcoholic liver disease
Sulfur metabolism
Glycosaminoglycan biosynthesis − heparan sulfate / heparin
Fatty acid biosynthesis
Lipoic acid metabolism
Nicotinate and nicotinamide metabolism
Taurine and hypotaurine metabolism
Phosphonate and phosphinate metabolism
Cholinergic synapse
−1.0
−0.5
0.0
0.5
1.0
Differential Abundance Score (DA Score)
Count
1
2
3
4
5
KEGG.class
Carbohydrate metabolism
Digestive system
Endocrine and metabolic disease
Energy metabolism
Glycan biosynthesis and metabolism
Lipid metabolism
Metabolism of cofactors and vitamins
Metabolism of other amino acids
Nervous system

C

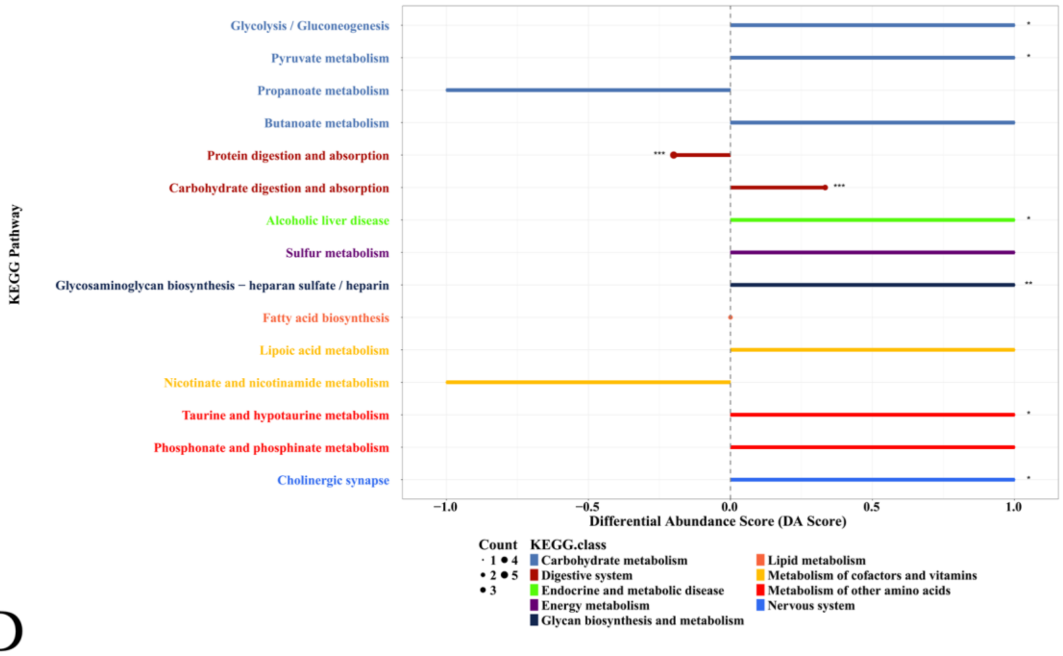
Glycolysis / Gluconeogenesis
Pyruvate metabolism
Propanoate metabolism
Butanoate metabolism
Protein digestion and absorption
Carbohydrate digestion and absorption
Alcoholic liver disease
Sulfur metabolism
Glycosaminoglycan biosynthesis − heparan sulfate / heparin
Fatty acid biosynthesis
Lipoic acid metabolism
Nicotinate and nicotinamide metabolism
Taurine and hypotaurine metabolism
Phosphonate and phosphinate metabolism
Cholinergic synapse
KEGG Pathway
−1.0
−0.5
0.0
0.5
1.0
Differential Abundance Score (DA Score)
Count
1
2
3
4
5
KEGG.class
Carbohydrate metabolism
Digestive system
Endocrine and metabolic disease
Energy metabolism
Glycan biosynthesis and metabolism
Lipid metabolism
Metabolism of cofactors and vitamins
Metabolism of other amino acids
Nervous system

D

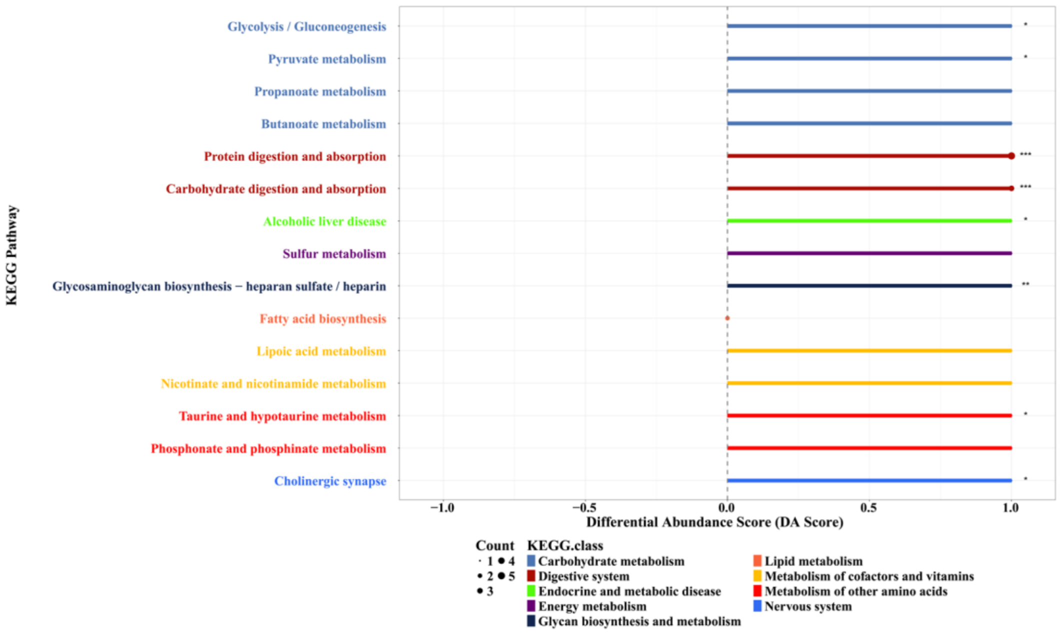
Glycolysis / Gluconeogenesis
Pyruvate metabolism
Propanoate metabolism
Butanoate metabolism
Protein digestion and absorption
Carbohydrate digestion and absorption
Alcoholic liver disease
Sulfur metabolism
Glycosaminoglycan biosynthesis − heparan sulfate / heparin
Fatty acid biosynthesis
Lipoic acid metabolism
Nicotinate and nicotinamide metabolism
Taurine and hypotaurine metabolism
Phosphonate and phosphinate metabolism
Cholinergic synapse
KEGG Pathway
−1.0
−0.5
0.0
0.5
1.0
Differential Abundance Score (DA Score)
Count
1
2
3
4
5
KEGG.class
Carbohydrate metabolism
Digestive system
Endocrine and metabolic disease
Energy metabolism
Glycan biosynthesis and metabolism
Lipid metabolism
Metabolism of cofactors and vitamins
Metabolism of other amino acids
Nervous system

E

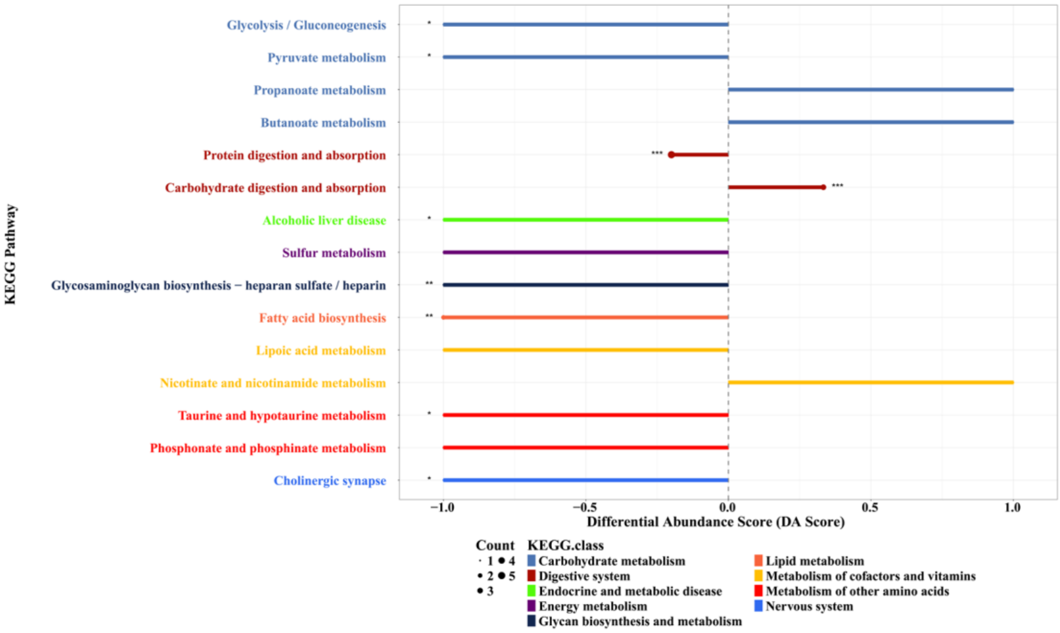

Figure S1. KEGG pathway enrichment differential analysis: (**A**) CK group versus RO group, (**B**) CK group versus CA group, (**C**) CK group versus SO group, (**D**) CK group versus HSO group, and (**E**) CK group versus LSO group (* $P < 0.05$, ** $P < 0.01$, *** $P < 0.001$).
